# The Role of Immersive Virtual Reality in Upper Limb Rehabilitation for Subacute Stroke: A Review

**DOI:** 10.3390/jcm14061903

**Published:** 2025-03-12

**Authors:** Danilo Donati, Elena Pinotti, Monica Mantovani, Silvia Casarotti, Annalisa Fini, Roberto Tedeschi, Serena Caselli

**Affiliations:** 1 Rehabilitation Medicine, Department of Neuroscience, Azienda Ospedaliero-Universitaria di Modena, 41125 Modena, Italy; danilo.donati@unimore.it (D.D.); pinottielena01@gmail.com (E.P.); mantovani.monica@aou.mo.it (M.M.); casarotti.silvia@aou.mo.it (S.C.); fini.annalisa@aou.mo.it (A.F.); caselli.serena@aou.mo.it (S.C.); 2 Clinical and Experimental Medicine PhD Program, University of Modena and Reggio Emilia, 41121 Modena, Italy; 3 Department of Biomedical and Neuromotor Sciences, Alma Mater Studiorum, University of Bologna, 40126 Bologna, Italy

**Keywords:** motor impairments, immersive virtual reality (VR), upper limb rehabilitation, subacute stroke

## Abstract

**Background:** Patients with stroke sequelae experience motor impairments that make it difficult to perform many activities of daily living, resulting in reduced social participation. Immersive virtual reality (VR) provides the necessary conditions for motor learning, such as repetitiveness, intensity, and task meaningfulness, and it could be a promising rehabilitation tool for upper limb recovery in individuals with stroke sequelae. **Objective:** The objectives of this study are to summarize the current scientific evidence on the use of immersive VR for upper limb rehabilitation in patients with subacute stroke and to identify clinical and instrumental criteria that may inform the development of a standardized VR treatment protocol. **Materials and Methods:** Bibliographic research on primary and secondary studies was conducted using the keywords “subacute stroke”, “immersive virtual reality/head-mounted display (HMD)”, and “upper extremity/arm/hand” in the following electronic databases: CINAHL, PubMed (MEDLINE), Embase, Web of Science, Cochrane Library, PEDro, and Google Scholar. Then, we performed the selection of studies and the assessment of the methodological quality of such studies using the PEDro scale. Finally, the qualitative synthesis of the data extracted from the selected studies was carried out. This systematic review was conducted according to the PRISMA 2020 guidelines. **Results:** After the selection process, five studies were included in this systematic review (two RCTs, two controlled clinical studies, one study protocol). Four studies reported significant improvements in some main outcomes after the VR intervention, including a significant increase in the Fugl-Meyer Upper Extremity total score, in favor of the virtual rehabilitation group. **Conclusions:** VR appears to be a promising rehabilitation tool for upper limb motor recovery. However, further research is needed to determine the intervention methods and long-term effects of VR on the stroke population.

## 1. Introduction

Stroke is a cerebrovascular disease, and it represents the second leading cause of death and the third leading cause of disability worldwide [1].

Stroke survivors manifest motor impairments in 80% of cases, which include alterations in muscle tone, weakness, and the loss of coordination, resulting in less motor control in the trunk and upper and lower limb movements. These symptoms can be associated with dysphagia and disability in terms of language, sensory, behavioral, visual, and cognitive functions (difficulty in intellectual capacity, memory, attention, orientations, awareness) [2,3]. The paralysis or paresis of the upper limbs represents the most frequent onset sign of stroke. These disorders make it difficult to perform many activities of daily living, especially those that depend on the coordination of both upper limbs or require fine hand movements. The resulting loss of activity tends to reduce the individual’s social participation. Therefore, improving upper limb function is a core element of rehabilitation after stroke to maximize recovery [4,5]. Several animal studies demonstrate that the subacute period represents the most critical phase for regaining motor skills. This period is a window of enhanced neuroplasticity, during which the brain’s dynamic response to injury is heightened and rehabilitation might be particularly effective [6]. A specific rehabilitation program in the subacute phase is a key element in preventing long-term disability that would affect the quality of life of patients [7]. In recent years, alongside traditional rehabilitation techniques, new technologies such as virtual reality (VR) have been developed to promote neuromotor reorganization and reduce motor impairment [8]. Several randomized clinical trials (RCTs) have shown that virtual reality therapy (VRT) significantly facilitates the motor and functional recovery of the upper limbs, gait, balance, health-related quality of life, and activities of daily living when used alongside conventional therapy. However, VR has not been shown to be consistently superior to conventional therapy alone. Its specific underlying mechanisms remain elusive at this stage [9].

Salzer et al. defined virtual reality as “the use of interactive simulations to provide the users with opportunities to engage in environments that appear and feel similar to real-world objects and events” [10]. In other words, it is a technology that allows users to interact with a simulated environment and receive feedback on their performance in real time.

Immersion is a key concept of virtual reality. It refers to the extent to which users feel present being in a virtual environment rather than in the real world, and it is correlated with the technology used by a system [11,12]. Technologies range from fully immersive applications to non-immersive applications, progressing through semi-immersive ones.

With immersive virtual reality, the user experiences the sensation of being completely “immersed” in a computer-generated virtual environment [13]. Devices using this technology have some tracking sensors that provide real-time images with a visual perspective that changes according to the user’s head and body movements. The images are often presented through a head-mounted display (HMD). An HMD has two small displays (positioned near the eyes, inside goggles or a helmet), a head tracking sensor (to adapt the displayed images to head movements), and a headphone for auditory signals. The user only sees the three-dimensional image generated by the computer, and the rest of the physical world is excluded from view. For full immersion, immersive VR systems, in addition to visual and auditory devices, often employ tactile devices, such as gloves, which are used as both motion tracking sensors (and to issue commands) and as devices capable of providing tactile feedback to the user in real time.

This type of VR utilizes scenarios designed based on principles that guide neuroplasticity (the neurobiological process underlying the recovery of cognitive and motor functions) and facilitate motor learning: the intensity, frequency, and repetition of exercise; an enriched environment with multisensory stimuli; the quality of feedback; patient motivation; and the significance of the task required [13].

The distinctive features of the virtual environment created by the immersive system and the multiple sensory interactions between the subject and the simulator allow the user to perceive the virtual scenario as a realistic experience, enhancing the patient’s ability to transfer the skills learned in the virtual environment to real life [14,15]. The virtual tasks proposed to the patient are perceived as more interesting and enjoyable compared to those performed in a normal rehabilitation context, increasing the patient’s motivation. This characteristic encourages the repetition of the required exercise, which is a key factor in motor learning [15].

A non-immersive virtual environment is commonly experienced in two dimensions and is delivered through a computer display or a gaming system for consoles [16]. The user can interact with the environment shown on a screen using tools such as a mouse or a joystick. The perspective is allocentric (third-person), and an avatar is displayed on the monitor [17]. These systems include all basic forms of gaming devices such as Playstation, Xbox, and computers.

A semi-immersive system is in the middle between an immersive and non-immersive system. Using a computer screen or VR googles, the user can visually explore the virtual environment but will not perceive other types of physical sensations. This means that a semi-immersive virtual reality allows the user to be in a different reality while still being connected to the physical, real environment.

Studies on virtual reality show significant heterogeneity in terms of the following: sample size, types of patients considered (pathology, distance from the lesion), types of virtual reality, devices used to deliver virtual reality treatment, protocol for administering virtual reality treatment, outcomes considered, and the presence and type of the control group. Therefore, the applicability of virtual reality for upper limb motor recovery in stroke patients needs further investigation to clarify the best approach to adopt and the most suitable technology to use for this patient population.

The aim of this review is to summarize the scientific evidence supporting the use of immersive virtual reality in upper limb rehabilitation for patients with subacute stroke. Another objective is to identify the presence of clinical and instrumental criteria useful for defining an evidence-based rehabilitation treatment protocol with virtual reality in the rehabilitation of the upper limbs in stroke patients.

## 2. Materials and Methods

This systematic review was conducted and reported in conformity with the PRISMA statement (Preferred Reporting Items for Systematic Reviews and Meta-Analyses) [18].

### 2.1. Eligibility Criteria

We defined the following eligibility criteria for the subsequent inclusion of studies:

1. Studies with samples of subjects with subacute stroke sequelae (period from 7 days to 6 months following the occurrence of stroke) [19];
2. Studies which applied immersive virtual reality as a rehabilitation strategy;
3. Studies considering upper limb motor recovery as the outcome.

### 2.2. Search Strategy

We explored 7 electronic databases (identification process): CINAHL, PubMed (MEDLINE), Embase, Web of Science, Cochrane Library, PEDro and Google Scholar.

To ensure a comprehensive review, we also included the gray literature and preprints by searching repositories such as OpenGrey, arXiv, and medRxiv. This approach was used to aim to capture additional relevant studies that might not have undergone peer review yet, providing a broader perspective on the topic.

The clinical question that led to undertaking this systematic review was the following: “What is the scientific evidence supporting the use of immersive virtual reality in the rehabilitation of the upper limb in stroke survivors in the subacute phase?” (where the subacute phase is defined as a period from 7 days to 6 months following the occurrence of stroke) [19].

From this question, we extracted the following P.I.C.O.S. (Population, Intervention, Comparison, Outcome, Study design):

P: Subacute stroke.

I: Immersive virtual reality/head-mounted display—HMD.

C: (not defined, other rehabilitation or sham treatment).

O: Upper extremity/hand/arm.

S: (not defined).

These terms were integrated using Boolean operators AND and OR in the databases examined.

No time limits were applied regarding the publication date of the studies, but the English language limit was imposed.

The search strings used are described in detail in Table 1.

### 2.3. Selection of Studies

**Figure 1:**
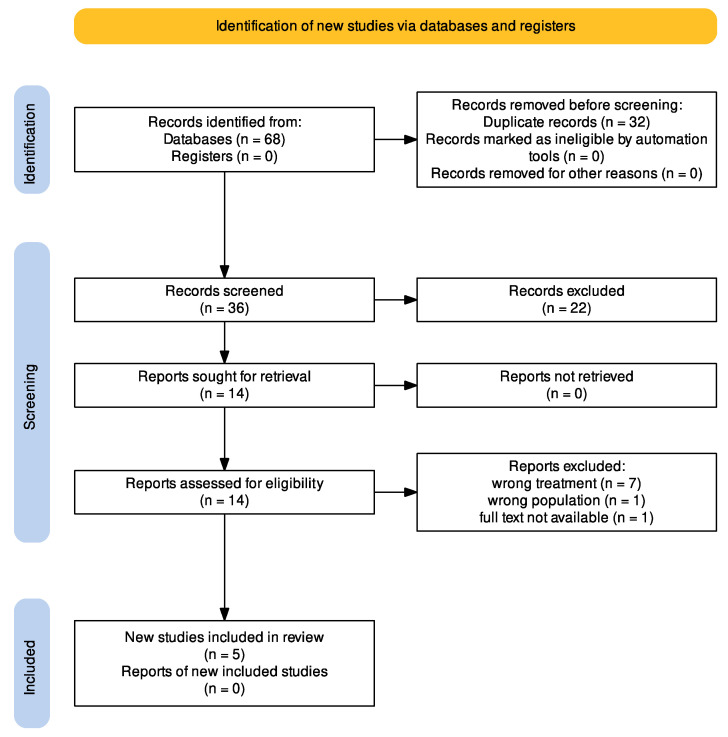
PRISMA 2020 Flow Diagram.

The selection of studies was carried out by two independent reviewers from the working group, following the screening, eligibility, and inclusion process after the identification process described above. The results obtained from the literature search were imported into the Rayyan web app [20], which allowed for the identification and removal of duplicates. For the remaining studies, the screening of titles/abstracts was conducted, excluding those that were not relevant. The eligibility process was carried out by reading the full text: studies that did not meet the inclusion criteria were excluded. The remaining studies were included in this review. At the end of each process, there was a discussion between the two reviewers to reach an agreement on any selection conflicts. To ensure reliability, the inter-reviewer agreement for study selection was assessed using Cohen’s kappa coefficient. A κ value of 0.04 was obtained, indicating poor agreement between reviewers. Any discrepancies were resolved through discussion. The entire process is outlined in the PRISMA Flow Diagram (Figure 1).

### 2.4. Methodological Quality Assessment

The methodological quality of the randomized controlled trials (RCTs) and controlled clinical studies included in this review was assessed using the PEDro scale (Physiotherapy Evidence Database Scale) [21]. It is used to evaluate the internal validity, clinical relevance, and external validity of RCTs.

The PEDro scale consists of 11 items, each of which, if fully satisfied, is awarded one point, for a total score ranging from 0 to 10, as the first item is not included in the final score. Among the items, the first refers to external validity, items two through nine consider the internal validity of this study, and the last two provide information regarding the interpretability of the results.

Many authors consider studies with a total score of 0 to 3 on the PEDro scale to be of low quality, a score of 4–5 as fair quality, a score of 6 to 8 as good quality, and a score of 9–10 as excellent quality. However, it is important to note that these classifications have not been validated [22]. Additionally, given the nature of RCTs with virtual reality intervention, it is not possible to blind patients and clinicians administering the intervention. Thus, a score of 8 on the PEDro scale is considered the highest possible score. The PEDro scale was chosen over other tools, such as the Cochrane Risk of Bias Tool, due to its specific focus on clinical trials in rehabilitation and physiotherapy. It provides a standardized assessment of methodological quality and is widely used in stroke rehabilitation research. Unlike broader tools, the PEDro scale allows for a practical evaluation of essential study elements, such as blinding, allocation concealment, and intention-to-treat analysis, which are critical in rehabilitation trials.

### 2.5. Data Extraction and Analysis

Finally, a qualitative data analysis was conducted by comparing the selected studies. The following information was extracted from each study: authors and year of publication, study design, patient characteristics (sample size, patient gender, patient age, time since acute event, affected side), exclusion criteria, devices used, control group and experimental group with their respective treatments, outcomes measured, assessment scales used, and study results.

## 3. Results

### 3.1. Search Results

The initial search yielded 68 articles. After removing duplicates using the Rayyan app, 32 articles were screened. Selection based on the title and abstract allowed us to exclude another 22 articles, while the remaining 14 were considered relevant for further evaluation based on full-text reading. Then, seven records were excluded for using the wrong treatment, one for including the wrong population, and one for the unavailability of the full text. Finally, five studies were included in this systematic review. The entire process is represented in a PRISMA Flow Diagram (Figure 1).

The methodological quality of the studies included (except the study protocol) was assessed through the PEDro scale by a single independent reviewer. The results are reported in Table 2.

### 3.2. Description of Studies

#### 3.2.1. Synthesis of Results

In this review, we included five studies: two randomized controlled trials (RCTs) [23,24], two controlled clinical trials [25,26], and one study protocol [27], which will be described later.

The data extracted from the included studies are reported in Table 3, which concerns the characteristics of the studies, and in Table 4, which describes in detail the treatment reserved for the experimental group and the control group. All studies were conducted in China, while the study protocol will be conducted in Spain. Four studies included healthy subjects. The controlled clinical trial of Mekbib et al. [25] did not include healthy subjects for the purpose of administering any treatment to them: they were included to compare their rs-fMRI (resting state functional magnetic resonance imaging) data with those of the patients of an experimental group at the end of the VR intervention. In Long et al.’s [26] study, healthy subjects underwent treatment with VR to compare their data with those of stroke patients.

Mekbib et al.’s RCT [23] also included healthy subjects (in addition to the experimental and control groups) to compare their rs-fMRI data with those of the patients of an experimental group at the end of the VR intervention. Finally, Huang et al. [24] also included healthy subjects (in addition to the experimental and control groups) to compare their rs-fMRI data with both those of an experimental group and those of a control group.

The two studies of Mekbib et al. [23,25] included patients within 3 months of stroke, while Huang et al. [24] included patients within 1 month of stroke. It was only in the study of Long et al. [26] that not all patients involved were in the subacute phase: five patients were in the chronic phase, while five patients were in the subacute phase, with an average of 15 months from stroke.

Regarding the severity of stroke, in the two studies of Mekbib et al. [23,25], the included patients had upper limb impairments ranging from moderate to severe. Huang et al. [24] included patients classified in the second, third, or fourth Brunnstrom stage. In the study of Long et al. [26], patients had to have a minimum score of 35 on the Fugl-Meyer Upper Extremity (FM-UE) scale to be included in the study.

The sample size of the studies was relatively small, ranging from 8 to 21 participants in the groups. The duration of the intervention was similar for all studies, ranging from 2 to 3 weeks, but only Huang et al. [24] conducted a follow-up at 12 weeks after the end of the intervention.

Regarding the entirety of the intervention, Huang et al. [24] reported four subjects as lost (two per group) at the end of the intervention (at the third week) and another eight subjects lost (four per group) at the follow-up (at the fifteenth week). Mekbib’s RCT [23] lost five patients during the study for personal reasons. Additionally, the Barthel Index (BI) scores of one patient from the experimental group and one from the control group were excluded from the mean calculation, as well as the FM-UE score of one patient from the control group, due to extreme scores. Furthermore, four patients from the experimental group and two healthy subjects were excluded from rs-fMRI data collection. The controlled clinical trial of Mekbib et al. [25] reported the loss of four patients after the VR intervention, without being able to acquire their functional magnetic resonance imaging data. In Long’s study [26], three patients left due to the feeling of discomfort during treatment.

#### 3.2.2. The Aims of the Studies

The main objective of Mekbib et al. [25] in their controlled clinical trial was to use the MNVR-Rehab system (Mirroring Neuron VR Rehabilitation) as an immersive virtual rehabilitation tool based on Limb Mirroring Therapy (LMT), in order to stimulate the mirror neurons of the damaged areas and consequently facilitate the functional recovery of the upper limbs in stroke patients in the subacute phase with moderate/severe limb impairments.

The main objectives of Mekbib et al. in their RCT [23] were still to develop a system based on immersive virtual reality that could stimulate and activate mirror neurons (MNVR-Rehab in LTM mode) and also to use both upper limbs together (ALT mode) so that the affected limb could adopt the spatial and temporal characteristics of the unaffected limb in order to facilitate recovery after stroke in patients with moderate/severe limb impairments. The MNVR-Rehab system used in the two studies just described also emerges as a task-specific tool, which, according to the authors, encourages task repetitiveness.

Huang et al. [24] aimed to assess the effectiveness of immersive virtual reality rehabilitation in patients with subacute stroke and explore changes in brain connections as a result of this treatment. The VR treatment involved functional and meaningful tasks for the subjects, which could be repeated without causing fatigue and pain.

Finally, Long et al. [26] previously demonstrated that healthy subjects are able to transfer coordination skills to their non-dominant hand by performing a unimanual task with their dominant hand and visualizing a bimanual action through an HMD. In the study included in this review, the authors also proposed this task to patients with stroke to verify whether the coordination skills of the non-affected hand could be transferred to the paretic hand.

#### 3.2.3. The Intervention Protocols of the Experimental Group

Regarding the devices used in the experimental group, all studies used an HMD connected to two tracking stations *(HTC Vive*). In both of Mekbib’s studies [23,25], *Leap Motion* was used to track upper limb movements. This tool allows the patient to interact with the virtual scenario without using a joystick or controller, which could be too heavy to hold with the affected hand. Additionally, if the patient does not have enough strength to flex and extend the fingers of their hand to grab a ball, the system automatically attaches the ball to the virtual hand, without the patient noticing. This allows patients with significant upper limb impairments to still use the virtual system, which is called Mirroring Neuron VR Rehabilitation (MNVR). Long et al. [26] used gloves (*Hi5 VR gloves*) to track hand movements. Huang et al. [24], in addition to an HMD, also included auditory and tactile devices, but the authors did not specify the names of these tools.

The tasks that patients were required to perform were varied. The interventions are described in detail in Table 4.

#### 3.2.4. Outcomes

Only Huang et al. [24] performed an intention-to-treat (ITT) analysis. In both included RCTs [23,24], the clinical effects of the treatment were measured using the Fugl-Meyer Upper Extremity (FM-UE) scale and the Barthel Index (BI). In the study of Huang et al. [24], the scales were administered before the intervention, after the intervention (3 weeks), and after 12 weeks of follow-up, while in Mekbib’s RCT [23], the data were collected before and after the intervention with VR.

In Mekbib’s RCT, the experimental group reported significant improvements in both the FM-UE score (*p*-value = 0.0001) and the BI score (*p*-value = 0.003) after treatment with VR compared to the baseline. Furthermore, a significant difference was found between the experimental and control groups in the FM-UE score (*p*-value = 0.007, Cohen’s d = 0.7 (effect size)) after the intervention, while no significant difference was observed between the two groups in terms of the BI (*p*-value = 0.193).

In the study of Huang et al. [24], the intention-to-treat (ITT) analysis showed that the FM-UE score had significantly improved in the experimental group compared to the control group both at the post-intervention assessment (mean difference between groups = 9.11, 95% CI, *p*-value = 0.019) and at follow-up (mean difference = 11.47, 95% CI, *p*-value = 0.020). These results are consistent with the per-protocol (PP) analysis both after the intervention (mean difference = 12.46, 95% CI, *p*-value = 0.003) and at follow-up (mean difference = 18.85, 95% CI, *p*-value = 0.006). Similarly, the BI score had also significantly improved in the experimental group compared to the control group both after the intervention (for ITT, mean difference = 8.28, 95% CI, *p*-value = 0.048; for PP, mean difference = 9.77, 95% CI, *p*-value = 0.038) and at follow-up (for ITT, mean difference = 4.81, 95% CI, *p*-value = 0.019; for PP, mean difference = 6.69, 95% CI, *p*-value = 0.034).

Finally, in the experimental group, a significant improvement was observed between baseline and post-intervention regarding the FM-UE score (*p*-value = 0.002). Conversely, concerning the FM-UE score, no significant improvement was observed in the experimental group between post-intervention and follow-up (*p*-value = 0.303), despite a continuous increase in the score of this scale.

Resting state fMRI was used in the last two studies described to evaluate the neurobiological correlations of rehabilitation with VR and to find a correlation between changes in brain activity and motor recovery. In Mekbib’s RCT [23], the rs-fMRI data of subjects undergoing VR treatment were compared with those of healthy subjects involved in the study (to whom no intervention was applied). In healthy subjects, in the dominant hemisphere, the primary motor cortex was functionally connected with widespread bilateral brain regions, while the patients’ brain before the VR intervention showed abnormal functional connectivity (the lesioned motor cortex remained connected only with intra-hemispheric regions). After the intervention, the ipsilesional primary motor cortex was functionally connected with other sensorimotor areas, such as the contralateral primary motor cortex, the bilateral primary sensory cortex, and the contralesional superior frontal gyrus (*p*-value < 0.001). The study also showed that areas such as the primary motor and sensory cortices, the superior parietal gyrus, the cerebellum, and the supplementary motor area demonstrated substantial alterations after treatment, indicating that the MNVR-Rehab system had indeed addressed these MN-relevant areas.

Huang et al. [24] compared the rs-fMRI data of the experimental group with those of the control group in three different phases: before the intervention with VR, at the end of the intervention (3 weeks), and at the follow-up (12 weeks after the end of the intervention). At the end of the VR intervention (after 3 weeks) in the experimental group, the areas where a greater increase in brain connectivity was observed were the ipsilesional dorsal premotor cortex (*p*-value = 0.008) and the ipsilesional primary motor cortex (*p*-value = 0.003). These areas are associated with the sensory/motor network of the hand and had various connections with the ventral and dorsal attention networks related to attention, the fronto-parietal network, and the cingulo-opercular network. The authors deduced that the unique characteristics of the immersive virtual system, encouraging repetitive functional movements and stimulating attention, may have enhanced planning, motor learning, and motor control.

This phenomenon is more evident in the ipsilesional dorsal premotor cortex, where a change in functional connectivity has been associated with a significant improvement in upper limb motor performance (FM-UE) (Pearson correlation coefficient r = 0.484, *p* = 0.020) and activities of daily living (BI) (r = 0.575, *p* = 0.004). In the control group, on the other hand, the ipsilesional (*p*-value = 0.003) and contralateral (*p*-value < 0.001) dorsolateral prefrontal cortices were particularly active. These areas belong to the DMN (Default Mode Network), which is a set of cortical networks active at rest. In the intervention group, the DMN was deactivated during some task-oriented activities. According to the authors, treatment with an immersive virtual system may have contributed to a deeper involvement, resulting in lower functional connectivity to the DMN.

At the follow-up (12 weeks after the end of the intervention), the authors found other areas that differed between the two groups, again in terms of brain connections. In the experimental group, three areas of the visual network (bilateral primary visual cortex and ipsilesional lateral occipital cortex) developed different connections with areas of the contralateral cortex, such as the sensory/motor network of the hand, the visual and auditory network, the dorsal and ventral attention networks, and the cingulo-opercular network. This means that, compared to the control group, the immersive virtual system, 5 months after the intervention, may have allowed for more connections from the visual network to the sensory/motor system of the hand and the cingulo-opercular network in the contralateral cortex, enhancing a compensatory mechanism for activities lost on the ipsilesional side. Another area particularly active in the experimental group was the contralateral superior parietal gyrus, a center for the exchange of sensory and motor information, crucial for guiding upper limb movements towards the target and regulating hand grip to grasp the target. Additionally, the areas that showed an increase in brain connectivity post-intervention (3 weeks), associated with an improvement in the FM-UE score, had returned to the same level as the baseline at follow-up. This means that these three regions were specifically influenced during the rehabilitative intervention. The authors therefore concluded that rehabilitation with VR not only improved the functional activity of the injured hemisphere immediately after treatment but also supported the motor recovery of the affected upper limb by reorganizing brain activity in the contralateral cortex at a later stage (12 weeks after the intervention).

In the controlled clinical study of Mekbib et al. [25], the FM-UE score was the primary outcome. The authors observed a statistically significant improvement in the score of this scale after the VR intervention (*p*-value < 0.042, Cohen’s *d* = 0.7, moderate effect).

This study also compared the data from the rs-fMRI of 13 healthy subjects with those of stroke patients. In healthy subjects, many bilateral areas were connected to the primary motor cortex of the dominant hemisphere. In patients before RV treatment, the ipsilesional primary motor cortex had predominantly intra-hemispheric connections. After treatment, the connections between the ipsilesional primary motor cortex and contralateral regions related to motor control and sensory information processing were more explicit.

Furthermore, a significant direct positive correlation (coefficient of determination (R^2^) = 0.729, *p*-value < 0.037) was observed between changes in inter-hemispheric brain connections and FM-UE values.

Long et al. [26] considered several parameters as outcomes: total time to draw, reaction time, temporal asynchrony between the two hands, the intramanual spatial measure of the non-dominant/paretic hand (calculating the angle between two successive segments; for a perfect performance, this angle should be 90°), and the intermanual spatial measure (calculating the absolute angular difference between the segments generated by the paretic/non-dominant hand and the analogous segments generated by the non-paretic/dominant hand). These data were collected during the first phase of the intervention and during the third phase (pre/post-training), in which the subject had to use both hands to draw. At the evaluation performed in the third phase, all parameters decreased significantly compared to the first phase, both in the group of healthy subjects and in the group of stroke patients. These results allowed the authors to conclude that the spatial and temporal accuracy of the movements of the paretic hand can be improved through the unilateral training of the unaffected hand while receiving sensory/visual feedback as if both hands were involved in the training.

Furthermore, in this study, EEG was used, and it showed improvements in cortical networks in terms of their ability to process local information and an increase in efficiency in terms of processing global information after the intervention.

#### 3.2.5. Study Protocol

This research also identified a study protocol [27], which we considered but did not include in this review, as the results are not yet available. This study protocol is a single-blind randomized controlled trial (RCT) aimed at verifying the effects of the NeuRow system. It is a multimodal immersive Brain–Computer Interfacing (BCI) system based on motor imagery but using virtual reality. Subjects in the experimental group will receive the NeuRow treatment immediately after undergoing bilateral repetitive transcranial magnetic stimulation (rTMS). Their data will be compared with those obtained from the control group, which will only undergo repetitive transcranial magnetic stimulation. Both groups will receive conventional rehabilitation treatment.

To be included in this study, patients must be at least 3 months post-stroke, and their FM-UE score must be greater than 25. The primary clinical outcomes will be assessed using the Motricity Index (MI), a dynamometer, the FM-UE scale, and the Stroke Impact Scale (SIS). Secondary clinical outcomes will be assessed using the Computerized Finger Tapping Task (FTT), Nine-Hole Peg Test (NHPT), Modified Ashworth Scale (MAS), Nottingham Sensory Assessment, and the Barthel Index (BI). To measure cortical changes, EEG and a TMS Resting Motor Threshold will be used. The assessments will be conducted at baseline, after two weeks of treatment, and after four weeks from the beginning of the intervention.

## 4. Discussion

### 4.1. The Interpretation of the Results

The aim of this review was to summarize the scientific evidence currently available in the literature supporting the use of immersive virtual reality in the rehabilitation of the upper limbs in patients with stroke outcomes in the subacute phase. This review also aimed to identify any clinical and instrumental criteria useful for defining an evidence-based rehabilitative treatment protocol with virtual reality in the rehabilitation of the upper limbs of these patients.

In this review, five studies were included, one of which was a study protocol. The results suggest that VR could be a promising tool in the rehabilitation of stroke patients. The population of interest in this review included patients with stroke outcomes in the subacute phase. Three studies included patients who were on average within a month and a half of the acute event; this time interval from the onset of stroke represents a relevant factor to consider, as some patients were in a more recent recovery phase than others. The study of Long et al. [26] represents an exception compared to the other studies described because it considered a sample composed of 50% of patients in the subacute phase, while the remaining part of the sample was in the chronic phase, and their results were not differentiated. This lack of distinction made it difficult to determine whether the intervention was more effective in chronic patients than in subacute ones.

In the study protocol [27], which we considered but did not include in this review, patients must be at least three months post-stroke. 

If we consider cognitive disorders, it may be interesting to note that almost all the studies explicitly excluded patients with these disorders. For example, in the RCT of Mekbib et al. [23], the authors excluded patients with “severe cognitive deficit” with an MMSE score lower than 16, while in Huang’s RCT [24], patients unable to understand the instruction given by the therapist due to severe cognitive disorders or aphasia were excluded. In the study of Long et al. [26], the authors simply mentioned “other neurological and/or orthopedic disorders” without explicit references to cognitive disorders. Finally, the study protocol of Sànchez-Cuesta [27] also excludes patients with unilateral spatial neglect, aphasia, or visual impairments. In summary, it can be inferred that VR seems to not be suitable for or has not yet been tested on samples of patients presenting such disorders.

Regarding the inclusion of patients with sensory impairments, in both studies of Mekbib et al. [23,25], patients with non-neurological visual and auditory impairments (unrelated to stroke) were excluded, and in the study protocol, patients with visual impairments will be excluded. In the remaining studies, this type of sensory impairment was not mentioned, but it would have been an important factor to consider, as visual/auditory problems could affect the patient’s performance when using VR. In fact, sensory disorders related to hearing and vision commonly compromise the ability of stroke patients to correctly interpret sensory information, affecting their ability to interact with the real world [28] and, consequently, also with the virtual world.

Some studies have also excluded patients with severe upper limb motor deficits (quantified in terms of the FM-UE score or Brunnstrom stage) but did not consider possible deficits in tactile and proprioceptive sensitivity, often present following a stroke. Such deficits can compromise posture and movement control, affect the quality of performance in motor tasks, and contribute to the slower recovery of motor activity [29,30,31,32,33]. Considering that proprioceptive disorders limit the performance of activities of daily living, as well as leading to longer hospital stays and, in general, worse outcomes [34], it would be important to understand how the presence of such disorders may affect the use of VR or the results of its application in stroke patient rehabilitation.

If we consider the control groups, great heterogeneity can be highlighted both in terms of subject characteristics and in terms of the interventions administered. In both included RCTs [23,24], the control group consisted of stroke patients who were only given conventional rehabilitation treatment. In the controlled clinical study of Mekbib et al. [25], however, the control group consisted of healthy subjects who did not receive any treatment, while their functional magnetic resonance imaging data were compared with those of the patients in the experimental group. In the study of Long et al. [26], the control group consisted of healthy subjects who received the same treatment as the experimental group. Finally, the control group in the study protocol [27] will consist of stroke patients who will only receive repetitive transcranial magnetic stimulation. Given the poor homogeneity among the control groups of the studies, it is difficult to draw precise and unequivocal conclusions about the real effectiveness of the treatment.

Regarding the immersive environment in which the participant was asked to perform the task, substantial differences emerge among the selected studies. In the studies of Mekbib et al. [23,25], the task simply involved picking up a ball and placing it inside a basket, while the intervention conducted by Long et al. [26] required patients to draw three-sided squares on a table. In the study protocol [27], on the other hand, patients will be asked to imagine using the oars of a boat.

The intervention closest to a setting of functional activities was that used by Huang et al. [24], where the subject was required to cook, fry food, or tidy up an office desk. Such activities certainly have a more concrete and practical application for the subject as they represent common activities in daily life.

In general, the settings proposed in almost all studies were characterized by “enriched” environments, with multisensory activities and rich feedback, but the significance of the task was lacking, as was the presence of an ecological and functional environment that encouraged task repetition and supported patient motivation [10,35,36,37,38,39].

All studies used immersive systems that allowed the patient to perceive and see themselves in the virtual environment in the first person, and all the activities proposed in the VR rehabilitation sessions required the patient to maintain a seated position. However, none of the included studies mentioned the need for a re-adaptation to the seated position. Head and trunk control plays a key role in supporting limb movement [40]. Therefore, it would have been useful to have information on the patients’ ability to maintain a seated position with adequate postural alignment and stability in order to better define the profile of patients suitable for VR rehabilitation treatment.

The rationale underlying the use of VR also varies depending on the study considered. Mekbib et al. [23,25] and Long et al. [26] used VR as a tool to provide mirror therapy-based treatment, while the study protocol by Sànchez-Cuesta et al. [27] is based on motor imagery. In the case of the study conducted by Huang et al. [24], however, the emphasis was on the intensity and repetitiveness of the task in terms of task-oriented exercises within a recreational and stimulating context, including some activities that simulate everyday life situations.

It can therefore be stated that the rationale behind the use of VR also represents another element of heterogeneity, which is reflected in the setting used and does not allow for the synthesis of results to draw unequivocal conclusions in order to create a VR administration protocol.

As for the outcome assessments, Mekbib et al. [23,25] and Huang et al. [24] adopted the Fugl-Meyer Upper Extremity (FM-UE) scale to measure upper limb structure and functions, while they used the Barthel Index (BI) as an activity scale.

Considering the FM-UE score, in both RCTs, a statistically significant difference was observed after the VR intervention in the experimental group compared to the control group. Furthermore, in both RCTs, the experimental group reported significant improvements in the score of this scale after the VR treatment compared to the baseline. However, Huang et al. [24] observed that between the post-intervention (3 weeks) and the follow-up (12 weeks after the end of the intervention) in the experimental group, there were no significant improvements in the FM-UE score. While the FM-UE scale provides a quantitative measure of motor recovery, it is crucial to assess whether these improvements translate into meaningful functional gains. Studies have shown that FM-UE improvements correlate with enhanced activities of daily living, but the degree of functional impact varies. Future trials should incorporate additional functional outcome measures, such as the Action Research Arm Test (ARAT) or the Wolf Motor Function Test (WMFT), to better quantify real-world benefits.

On the other hand, the BI showed a significant improvement in the experimental group compared to the control group only in the study of Huang et al. [24]. In Mekbib’s RCT [23], however, this index after the VR intervention significantly improved in the experimental group compared to the baseline, but no significant difference was observed between the two groups.

The lower ability of the BI to detect significant changes between the experimental and control groups may be related to the fact that this type of assessment includes items that evaluate activities in which the use of the upper limbs is poorly considered or not even expected. At the same time, the greater ability of the FM-UE scale to detect such changes may express an improvement in the motor functions of the upper limbs (e.g., strength, articulation, muscle tone), which, however, does not translate into an improvement in motor activities involving the upper limbs. In fact, numerous studies in the literature use the FM-UE scale in combination with other assessment scales that more accurately quantify the activity of the upper limbs [41], such as the *Frenchay Arm Test (FAT)*, the *Chedoke Arm and Hand Activity Inventory (CAHAI)*, the Action Research Arm Test (ARAT), and the Wolf Motor Function Test (WMFT). The studies of Mekbib et al. [23,25] and Huang et al. [24] revealed (after the intervention with VR) a greater functional connectivity between the ipsilesional primary motor cortex and the contralateral primary motor cortex, as well as with the bilateral sensory cortex and the contralateral superior parietal gyrus. This area is a key hub for the exchange of sensory and motor information essential for guiding upper limb movements towards the target and regulating hand grip to grasp the target [42]. Moreover, it is implicated in body movement control, visual movements, oculomotor nerve activity, and visual–spatial orientation [43,44,45,46,47]. Treatment with an immersive VR system may have strengthened the functions of the superior parietal gyrus, promoting an improvement in upper limb performance.

Based on the results obtained in this review and their discussion, it was possible to identify the following indications regarding clinical and instrumental criteria in order to develop an evidence-based treatment protocol with virtual reality for the recovery of the upper limbs in patients with subacute stroke:

- The immersive system used consist of an HMD device connected to HTC Vive motion tracking stations.
- VR treatment assumes an intensive nature, with sessions ranging from 30 to 60 min of use, 3 to 5 times a week, for a period of 2, 3, or 4 weeks.
- The use of VR is currently always integrated with conventional rehabilitation, so it must be considered as an additional therapy and not a substitute.
- It is necessary that the patient does not present either cognitive disorders (e.g., neglect, aphasia, dementia) or sensory disorders of an auditory or visual nature.
- The characteristics of patients eligible for VR treatment vary depending on the rationale underlying the use of VR. For example, if the rationale is related to motor imagery, the patient will simply have to imagine performing the task, standing still. Consequently, in this case, the necessary characteristics in terms of structural, functional, and postural aspects will differ from those expected for a patient undergoing VR therapy based on mirror therapy, who is required to move their limbs.

#### 4.1.1. Comparison of VR Modalities in Stroke Rehabilitation

While immersive VR offers a fully engaging environment that enhances sensorimotor feedback, semi-immersive and non-immersive VR provide alternative options that may be more accessible and feasible for certain populations. Immersive VR is preferable when deep engagement and enhanced neuroplasticity are required, particularly in patients needing intensive motor retraining. However, semi-immersive VR may be more suitable for patients with cognitive impairments or those prone to cybersickness. A detailed comparison is provided in Table 5, outlining the advantages and limitations of each VR modality.

#### 4.1.2. Effectiveness of VR Across Stroke Severity Levels

The effectiveness of VR interventions may vary depending on stroke severity. Patients with mild impairments may benefit from VR to refine motor control and coordination, while those with moderate impairments may use VR to regain functional movement patterns. For severe cases, VR can serve as an adjunctive therapy but may require additional assistive devices or adapted protocols. More research is needed to establish tailored VR interventions for different severity levels.

Future research should focus on high-quality RCTs assessing the long-term benefits of VR in stroke rehabilitation. While short-term improvements in motor function are well documented, the sustainability of these gains remains unclear. Studies should incorporate follow-up assessments beyond 12 weeks to determine whether VR fosters lasting neuroplastic changes and functional recovery.

### 4.2. Limitations

This review has some limitations. First, the samples of the selected studies were relatively small, ranging from 8 to 21 patients per group, which limits the generalization of the results. Furthermore, the studies of Mekbib et al. [23,25] and Long et al. [26] did not provide information on the calculation of the sample size.

Two studies [25,26] evaluated with the PEDro scale obtained a low score, highlighting a poor methodological quality. This is also due to the fact that these studies did not involve a true control group consisting of stroke patients but rather included a group of healthy subjects.

Many studies, except for Huang et al.’s [24], were limited to an assessment immediately after the intervention, without conducting a follow-up evaluation. Furthermore, the authors did not report information on any adverse effects manifested by patients after treatment with VR (cybersickness). Consequently, the long-term effects of virtual reality and its safety could not be adequately determined.

It would have been appropriate to further analyze the results obtained from resting state functional magnetic resonance imaging, comparing those of the experimental group with those of the control group. However, only Huang et al. [24] conducted this type of comparison, while the other studies compared the fMRI data of the experimental group with those of healthy subjects.

In addition, it should be noted that in the studies of Mekbib et al. [23,25], the application of VR therapy was influenced by decisions made by the physiotherapist, such as the choice of whether or not to assist the patient and the selection of the mode to use (e.g., unilateral or bilateral LMT mode or ALT mode). Consequently, there may have been differences in the administration of the intervention due to inter-operator variability. Financial and logistical barriers remain key challenges to the widespread implementation of VR in stroke rehabilitation. The high costs of immersive VR systems, including hardware, software, and maintenance, limit their accessibility in many rehabilitation settings. Additionally, the need for trained personnel and space for VR-based therapy further complicates its integration into routine clinical practice. Future studies should explore cost-effective solutions and reimbursement models to enhance VR adoption.

## 5. Conclusions

This systematic review allowed us to conclude that virtual reality appears to be effective in improving the motor functions (quantified with the FM-UE scale) of the upper limbs in patients with subacute stroke. However, we found numerous elements of heterogeneity among the studies, including the rationale behind the use of virtual reality, the types of control groups, intervention methods, and the type of virtual reality treatment administered, which did not allow for precise and unambiguous conclusions to be made about the actual effectiveness of the treatment. Finally, as a result of the previously highlighted heterogeneity, we could only identify general indications regarding clinical and instrumental criteria useful for developing an evidence-based rehabilitative treatment protocol with virtual reality. These indications concern the intensity of the treatment; the use of virtual reality as a complementary treatment to conventional physiotherapy; and targeting patients with highly selected motor, sensory, and cognitive characteristics.

### Practical Guidelines for VR Integration

To effectively integrate VR into stroke rehabilitation, clinicians should consider the following guidelines:

- Select the appropriate VR modality based on patient needs (e.g., immersive for intensive motor retraining, semi-immersive for moderate engagement, non-immersive for basic exercises).
- Ensure VR sessions align with conventional rehabilitation goals rather than replacing traditional therapy.
- Gradually introduce VR to prevent cybersickness and optimize patient adaptation.
- Use objective outcome measures (e.g., FM-UE scale, ARAT) to track progress and adjust therapy accordingly.
- Address financial and technical barriers by exploring cost-effective solutions, such as shared VR equipment and tele-rehabilitation options.

Based on the results obtained, it would be useful to conduct further randomized controlled studies with greater homogeneity in the treatment administered and more detailed outcome evaluations in terms of function (e.g., muscle tone, dexterity, sensitivity) and ones that are more specific to the upper limbs in terms of activity. This could allow for more precise and reliable results on the actual effectiveness of virtual reality treatment in the recovery of the upper limbs in these patients and provide more information on the motor and sensory variables of the upper limbs influenced by the treatment.

## Figures and Tables

**Table 1:** Search strings used in databases.

CINAHL	(stroke subacute) AND (immersive virtual reality OR hmd) AND (upper extremity OR arm OR hand)
PubMed	(subacute stroke) AND (immersive virtual reality OR hmd) AND (upper extremity OR arm OR hand)
Embase	(subacute stroke) AND (immersive virtual reality OR hmd) AND (upper extremity OR arm OR hand)
Web of Science	(subacute stroke) AND (immersive virtual reality or hmd) AND (upper extremity OR arm OR hand)
PEDro	Subacute stroke AND hmd AND arm Subacute stroke AND immersive virtual reality AND arm Subacute stroke AND hmd AND hand Subacute stroke AND immersive virtual reality AND hand Subacute stroke AND hmd AND upper extremity Subacute stroke AND immersive virtual reality AND upper extremity
Cochrane Library	(subacute stroke) AND (immersive virtual reality OR hmd) AND (upper extremity OR arm OR hand)
Google Scholar	(subacute stroke) AND (immersive virtual reality OR hmd) AND (upper extremity OR arm OR hand)

**Table 2:** Methodological quality assessment (PEDro scale).

Authors	Eligibility Criteria	Random Assignment	Concealed Allocation	Baseline Comparability	Subject Blinding	Clinician Blinding	Assessor Blinding	Adequate Follow-Up (>85%)	Intention-to-Treat Analysis	Between-Group Comparison	Point Measures and Measures of Variability	Total Score
Mekbib et al. [23]	yes	x	x	x			x			x	x	6/10
Huang et al. [24]	yes	x	x	x			x		x	x	x	7/10
Mekbib et al. [25]	yes									x	x	2/10
Long et al. [26]	yes							x		x	x	3/10

Notes: the study protocol (Sànchez-Cuèsta et al. [27]) was excluded from the methodological quality assessment.

**Table 3:** The characteristics of the studies included in this review.

Author, Year	Type of Study	Sample Size	Gender (M/F)	Age (Mean and Ds)	Days from Stroke (Mean and Ds)	Affected Limb (r/L)	Exclusion Criteria	Intervention	Main Outcome and Assessment Scale	Results
Mekbib et al. 2021 [23]	RCT	EG: 12	9/3	52 ± 13	36.92 ± 22.04	5/7	MMSE < 16; hearing and vision not preserved	MNVR-Rehab system in LMT and ALT mode + conventional rehabilitation	Resting state (Rs) MRI + BI + FM-UE	MNVR-Rehab in LMT and ALT mode is an encouraging rehabilitation tool that may increase upper limb function in subacute stroke subjects compared to conventional rehabilitation.
		CG: 11	8/3	61 ± 7	39.36 ± 18.08	4/7	-	Conventional rehabilitation	Rs-MRI + BI + FM-UE	
Huang et al. 2022 [24] *	RCT	EG: 20	13/7	63 ± 14	18.80 ± 8.44	10/10	History of TIA; failure of critical organs; history of neurosurgery or epilepsy; severe cognitive impairments or aphasia	Conventional rehabilitation + immersive virtual reality with HMD	Rs-MRI + BI + FM-UE	An immersive environment is a promising rehabilitation tool for improving upper limb recovery in subacute stroke subjects. This improvement is associated with a cerebral reorganization which occurs not only immediately after the VR intervention but also in later phases.
		CG: 20	11/9	65 ± 6	19.00 ± 6.64	7/13	-	Conventional rehabilitation (physiotherapy and occupational therapy)	Rs-MRI + BI + FM-UE	
Mekbib et al. 2020 [25]	Clinical controlled study	EG: 8	6/2	57 ± 4	38	5/3	Moderate/severe visual, auditory, or cognitive impairments	MNVR-Rehab system in LMT+ conventional rehabilitation	Rs-MRI before and after the intervention + FM-UE	The unilateral and bilateral mirroring exercise of the upper limbs in an immersive virtual environment can increase cerebral reorganization and lead to the better functioning of the upper limbs.
		CG: 13 healthy subjects	NR	55 ± 7	-	-	-	-	RS-MRI at the beginning (as a comparison)	
Long et al. 2022 [26]	Clinical controlled study	EG: 10	5/5	55 ± 12	450 (Ds not available)	7/3	FM-UE score < 35; other neurological or orthopedic disease; discomfort/pain during activity	Performing unimanual tasks with the non-paretic hand and visualizing bimanual action through an HMD	EMG+ total time, reaction time, temporal asynchrony, intra-hand and inter-hand measurements	Coordination skills can be transferred to the paretic hand after performing short-term training with the non-paretic hand, collecting sensory feedback as if both hands were drawing. This would contribute to increasing the efficiency of the cortical neural network in both healthy subjects and those with stroke.
		CG: 10 healthy subjects	7/3	23 ± 3	-	-	-	As above	As above	
Sànchez-Cuesta et al. 2021 [27]	Study protocol	EG: 21	NR	>18 years	>90 (Ds not available)	-	History of seizure or brain aneurysm; pacemakers, pumps, metal implants in the head; clinical instability; MAS > 3; other pre-existing neurological disease or cerebrovascular accidents with sequelae; neglect, aphasia, visual problems; Brunnstrom stage = 1; FM-UE score < 25	Conventional rehabilitation + bilateral rTMS + immersive multimodal BCI-VR training system NeuRow	MI + FM-UE + Dynamometry + SIS	Not yet available.
		CG: 21	ND	>18 years	>90 (Ds not available)	-	-	Conventional rehabilitation + bilateral rTMS	MI + FM-UE + Dynamometry + SIS	

Abbreviations and notes: EG = experimental group; CG = control group; BI = Barthel Index; FM-UE = Fugl-Meyer Upper Extremity; MI = Motricity Index; SIS = Stroke Impact Scale. LMT = Limb Mirroring Therapy; ALT = affected limb therapy; ND = not detected. The study protocol is highlighted in gray. * not yet peer-reviewed.

**Table 4:** The descriptions of the treatments reserved for the experimental group and control group of the studies included in this review.

Author and Year	Experimental Group		Control Group	
	Description	Duration	Description	Duration
Mekbib et al. 2021 [23]	This group received both conventional rehabilitation and MNVR therapy. The MNVR-Rehab system includes the following elements: (1) a head-mounted display (HMD) to fully immerse the patient in the virtual environment (VE); (2) two base stations (HTC Vive tracking stations) to track the patient’s exact position in 3D; (3) Leap Motion to track the patient’s upper limb movements and transfer them onto the virtual limbs; and (4) a high-performance PC running the software system to generate the virtual environment and record the patient’s actions. The subject was seated in a chair wearing the HMD, through which they saw a virtual table. The task was to grasp balls and release them into a basket. The therapist could choose the difficulty of the task and the training mode. (1) LMT (limb mirror therapy) modes: Unilateral LMT mode: The patient was required to grasp and release target balls into the basket using the unaffected limb, but in the VE, the affected limb appeared to move. Bilateral LMT mode: The patient was required to grasp and release target balls into the basket using both virtual limbs by controlling their movements using their intact UE. In the VE, the system automatically displayed a pair of virtual limbs. (2) ALT (affected limb therapy) modes: Unilateral therapy mode: The patient was required to use their more affected UE to grasp balls. Bilateral therapy mode: The patient was required to grasp balls with both limbs simultaneously. After the first session (20 balls), the therapist could set the next one by changing the task’s difficulty based on the patient’s performance. The therapist could assist the patient in the case of difficulty.	1 h rehabilitation with MNVR + 1 h conventional rehabilitation a day, for 4 days a week, for 2 weeks (total: 8 h of MNVR and 8 h of conventional rehabilitation).	This group received only conventional rehabilitation, which included daily living activities, balance control, gait training, weight shift, and distal and proximal UE functional movements.	2 h a day, for 4 days a week, for 2 weeks.
Huang et al. 2022 [24]	This group received both conventional rehabilitation and VR rehabilitation. The devices used were an HMD connected to HTC Vive-VR stations. Subjects had to complete 6 programs: (1) frying food in a kitchen; (2) popping balloons with a sword in a virtual fencing room; (3) punching dolls in a virtual boxing arena; (4) playing basketball on a virtual court; (5) putting eggs in a basket; and (6) tidying up a desk in a virtual office. All tasks were to be performed with the affected limb. In the early stages of rehabilitation, subjects could perform the task with the help of the unaffected limb.	30 min of conventional rehabilitation + 30 min of VR rehabilitation, for 5 days a week, for 3 weeks.	This group only received conventional rehabilitation (physiotherapy and occupational therapy), including grips and selective finger movements, gross movement, strength training, stretching, and training in activities of daily life.	1 h a day, for 5 days a week, for 3 weeks.
Mekbib et al. 2020 [25]	This group received both conventional rehabilitation and MNVR therapy. The MNVR-Rehab system includes the same elements described in the study by Mekbib et al. from 2021 [23], cited above. The task was to grasp balls and release them into a basket. The therapist could choose the difficulty of the task and the training mode based on the patient’s characteristics. The mode was the LMT: Unilateral LMT mode: The patient was required to grasp and release target balls into the basket using the unaffected limb, but in the VE, the affected limb appeared to move. Bilateral LMT mode: The patient was instructed to move their unaffected UE in order to coordinate the right-side and left-side virtual limbs together for the purpose of grasping the target ball and releasing it during training. After the first session (20 balls), the therapist could set the next one by changing the task’s difficulty based on the patient’s performance. The therapist could assist the patient in the case of difficulty.	1 h rehabilitation with MNVR + 1 h conventional rehabilitation a day, for 4 days a week, for 2 weeks (total: 8 h of MNVR and 8 h of conventional rehabilitation).	Since they were healthy subjects, they did not receive any treatment.	-
Long et al. 2021 [26]	This group received VR therapy. An HTC Vive head-mounted display (HMD) was used, along with Hi5 VR gloves to record hand movements and 32 electrodes placed on the head to record EEG data. The subject was seated in front of a table wearing the HMD, through which they could see a virtual table. The experiment involved the patient tracing three-sided squares drawn on the table’s surface with their fingers (one on the right, one on the left), and it consisted of 3 phases: (1) the subject had to use both hands simultaneously to trace the squares; (2) the subject had to use only the unaffected limb, but in the virtual environment, both limbs were displayed; and (3) the subject had to use both hands again to complete the task.	32 trials (with different combinations of three-sided squares) for each phase.	Healthy subjects received the same VR treatment. The data were used in a comparison with those of stroke patients.	32 trials (with different combinations of three-sided squares) for each phase.
Sànchez-Cuesta et al. 2021 [27]	This group will undergo conventional rehabilitation + bilateral repetitive transcranial magnetic stimulation (rTMS), and immediately after, they will receive treatment with immersive virtual reality (NeuRow). The NeuRow treatment is based on motor imagery theory. In the first phase, the subject will be asked to imagine rowing a boat, and their brain activity will be recorded using electrodes applied to the head (EEG). EEG data will be used to distinguish the imagined movements of the right hand from those of the left hand. Then, the subject will wear an HMD, and they will see their upper limbs and two oars. In their hands, they will have two controllers that will make them perceive tactile stimuli. The subject will be asked to imagine the movement of the corresponding hand based on the stimuli presented on the screen (for example, if they see the left hand moving on the screen, the patient will have to imagine moving their left hand). The aim of this task is to perform as many correct motor imagery sequences as possible within a certain period of time.	rTSM: 5 times a week for 2 weeks (total of 10 sessions). NeuRow: 30 min, 3 times a week, for 4 weeks (total of 12 sessions).	This group will receive bilateral rTMS.	5 times a week for 2 weeks (total of 10 sessions).

Notes: the study protocol is highlighted in gray.

**Table 5:** Comparison of VR modalities in stroke rehabilitation.

Feature	Immersive VR (HMD)	Semi-Immersive VR (Projection Screens)	Non-Immersive VR (Desktop/Gaming)
Level of Immersion	High	Moderate	Low
Sensorimotor Feedback	High—Real-time interaction	Moderate—Limited interaction	Low—Interaction via controllers
Cognitive Load	High	Moderate	Low
Risk of Cybersickness	High	Moderate	Low
Equipment Cost	Expensive	Moderate	Affordable
Suitability for Severe Cases	High—Effective for intensive motor retraining	Moderate—Suitable for structured therapy	Low—Limited effects on severe cases
Suitability for Mild Cases	Moderate—May be excessive for mild cases	High—Good balance between engagement and feasibility	High—Suitable for simple exercises
Feasibility in Clinical Settings	Moderate—Requires dedicated space and equipment	High—Easier integration into clinics	High—Widely available and easy to use
